# Identification of *in vivo* nonlinear anisotropic mechanical properties of ascending thoracic aortic aneurysm from patient-specific CT scans

**DOI:** 10.1038/s41598-019-49438-w

**Published:** 2019-09-10

**Authors:** Minliang Liu, Liang Liang, Fatiesa Sulejmani, Xiaoying Lou, Glen Iannucci, Edward Chen, Bradley Leshnower, Wei Sun

**Affiliations:** 1 Tissue Mechanics Laboratory, The Wallace H. Coulter Department of Biomedical Engineering, Georgia Institute of Technology and Emory University, Atlanta, GA USA; 2 Emory University School of Medicine, Atlanta, GA USA; 3 Department of Computer Science, University of Miami, Coral Gables, FL USA

**Keywords:** Aneurysm, Biomedical engineering

## Abstract

Accurate identification of *in vivo* nonlinear, anisotropic mechanical properties of the aortic wall of individual patients remains to be one of the critical challenges in the field of cardiovascular biomechanics. Since only the physiologically loaded states of the aorta are given from *in vivo* clinical images, inverse approaches, which take into account of the unloaded configuration, are needed for *in vivo* material parameter identification. Existing inverse methods are computationally expensive, which take days to weeks to complete for a single patient, inhibiting fast feedback for clinicians. Moreover, the current inverse methods have only been evaluated using synthetic data. In this study, we improved our recently developed multi-resolution direct search (MRDS) approach and the computation time cost was reduced to 1~2 hours. Using the improved MRDS approach, we estimated *in vivo* aortic tissue elastic properties of two ascending thoracic aortic aneurysm (ATAA) patients from pre-operative gated CT scans. For comparison, corresponding surgically-resected aortic wall tissue samples were obtained and subjected to planar biaxial tests. Relatively close matches were achieved for the *in vivo*-identified and *ex vivo*-fitted stress-stretch responses. It is hoped that further development of this inverse approach can enable an accurate identification of the *in vivo* material parameters from *in vivo* image data.

## Introduction

Accurate identification of *in vivo* nonlinear, anisotropic mechanical properties of the vessel wall of individual patients has long been regarded as one of the critical challenges in the field of cardiovascular biomechanics^1^. Magnetic resonance imaging (MRI)^2^, ultrasound^3,4^ and computed tomography (CT)^5^ imaging techniques have been utilized to perform *in vivo* wall motion analyses. For example, MRI^2^ and ultrasound^3,4^ studies of ascending thoracic aortic aneurysm (TAA) patients have highlighted disparities between the dilated and non-dilated ascending aorta mechanics. However, such direct measurements of *in vivo* aortic wall deformation can only provide insight on TAA mechanical behaviors within the measured physiologic pressure range^5^ (usually between 80 mmHg–120 mmHg), whereas aneurysm rupture/dissection usually occurs under elevated arterial pressures (e.g., about 300 mmHg) brought on by extreme emotional or physical stress^6^. Thus, patient-specific TAA rupture analysis could benefit from estimating the full *in vivo* elastic properties using a constitutive model, which, consequently, can be utilized to predict TAA mechanical response at various loading conditions.

Recently, some studies^7,8^ derived linearized stiffness from *ex vivo* biaxial tests and showed that the stiff aneurysms are prone to rupture. This linearized metric can provide a simple and clinically-relevant way to roughly predict diameter/stretch-based rupture potential. The distribution of linearized stiffness has been measured on TAA from multiphase CT scans^9,10^. However, rupture analysis may benefit more from identification of nonlinear hyperelastic properties. For instance, the maximum curvature point of strain-stress curve has been shown to be the most important feature that is predictive of the aorta wall strength in machine learning models^11,12^. By extracting nonlinear properties from multiphase CT scans, it is feasible to derive intrinsic features such as the maximum curvature point, which may be used to estimate wall strength. Eventually, rupture risk prediction could be achieved by determining when the stress/stretch applied to the tissue exceeds its strength/extensibility^13^.

Since the unloaded state of arteries is unknown, it is challenging to inversely estimate hyperelastic constitutive parameters from *in vivo* deformed geometries. To simplify such inverse computation, the geometry of arteries is often assumed as a perfect tube. Based on this assumption, Schulze-Bauer and Holzapfel^14^ estimated Fung-type material parameters, Masson *et al*., Olsson and Klarbring, Stålhand^15–18^ estimated material parameters using the constitutive model proposed by Holzapfel *et al*.^19^ and geometrical parameters, Smoljkić *et al*.^20^ identified the Gasser–Ogden-Holzapfel (GOH) model^21^ parameters.

To account for the irregularity of patient-specific geometries, inverse finite element (FE) simulations are often used in the identification of *in vivo* hyperelastic properties from multi-phase clinical images. Optimization-based FE-updating approaches were proposed, in which the optimal set of material parameters is identified by updating the material parameters in the FE simulations to minimize a pre-defined error function. Using these strategies, Liu *et al*.^22^ estimated parameters of the modified Mooney-Rivlin model from carotid artery MRI data. The optimization problem can be much more challenging when estimating anisotropic model parameters, since different hyperelastic parameters are coupled nonlinearly in their contributions to the structural response. Wittek *et al*.^23,24^ developed two approaches to identify *in vivo* GOH model parameters of the abdominal aorta from 4D ultrasound data based on mixed stochastic-deterministic optimization. A total of 7400 iterations^23^ and 43,500–86,900 iterations^24^ were needed to reach the optimal set of parameters in their approaches, resulting in a computational time of 1~2 weeks. Such high computational cost could inhibit a practical use of the methods, particularly in a clinical setting requiring fast feedback to clinicians. To expedite the identification process, our group has recently proposed the multi-resolution direct search (MRDS) approach^25^, which was designed to improve the searching algorithm, and the computation time was reduced to 1~2 days with less than 1000 iterations. However, these studies^23–26^ relied on numerically-generated data to validate the approaches.

In this study, *in vivo* nonlinear anisotropic material properties of the aortic wall were estimated from clinical 3D gated CT images of two ascending thoracic aortic aneurysm (ATAA) patients. The MRDS approach^25^ was improved in this study in the following aspects: (1) without having to iteratively recover the unloaded configuration, the generalized prestressing algorithm (GPA)^27^ is implemented to directly account for the pre-stress state. Thus, the material parameter identification process is expected to be significantly accelerated; (2) rigid motions of the ascending aorta due to heart movements are removed using the rigid iterative closest point (ICP) registration algorithm^28,29^; and (3) to obtain the diastolic-to-systolic displacement field, non-rigid ICP registration^30^ and thin-plate spline (TPS) fitting^31^ algorithms are used to establish mesh correspondence between the two phases. For comparison, corresponding surgically-resected aortic wall tissue samples were obtained and subjected to planar biaxial tests to extract their experimentally-derived material properties. The estimated material properties were compared with the experimentally-derived material properties.

## Materials and Methods

### Image data and corresponding tissue specimens

**Figure 1:**
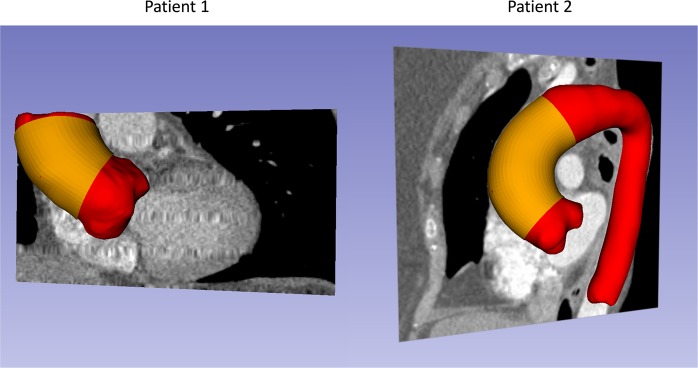
CT image segmentation of the aorta (red) and ATAA segment (yellow) for the two patients.

**Figure 2:**
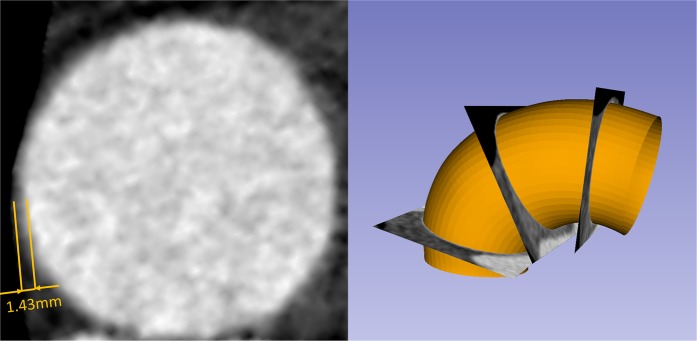
Measuring wall thickness from CT scans.

With Institutional Review Board (IRB) approvals, aortic tissue specimens from two patients (Patient 1: a 67 year-old male; Patient 2: a 68 year-old female) with ascending thoracic aortic aneurysm (ATAA) who underwent surgical repair was obtained from the Emory University Hospital, Atlanta, GA. The 10-phase preoperative ECG-gated CT data and systolic and diastolic blood pressure levels were obtained prior to the intervention. A complete waiver of HIPAA authorization and informed consent was granted by the Emory IRB. All data was collected retrospectively and de-identified and all methods were performed in accordance with the relevant guidelines and regulations. The CT images had a scan matrix size of 256 × 256, in-plane pixel size of 0.75 mm × 0.75 mm and slice thickness of 1 mm. Unfortunately, only part of ATAA of Patient 1 was imaged from the multiphase CT data. For each patient, the systolic and diastolic aorta geometries (Fig. 1, depicted in red) were reconstructed following our established protocol^32^. Wall thickness of the aorta can be obtained from high resolution CT images according to Shang *et al*.^33^. The thickness values were measured at 16 locations (Fig. 2) from cross-sectional planes of the ATAA segment (Figs 1 and 2, depicted in yellow) in the systolic phase. For each patient, surgically-excised aneurysmal tissue were dissected into 2~3 square-shaped specimens (2 specimens for Patient 1 and 3 specimens for Patient 2) for biaxial tensile tests. For one square-shaped specimen, the wall thickness values were measured at 3 equally-spaced locations along the diagonal line.

**Figure 3:**
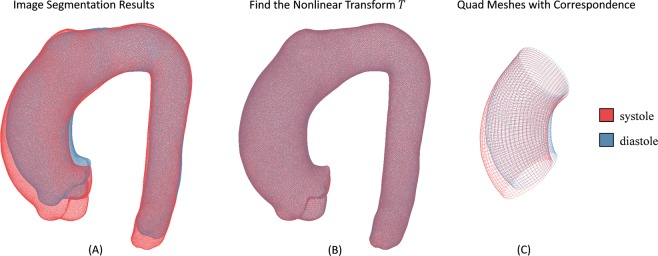
Surface registration and transform to establish mesh correspondence. (**A**) Diastolic geometry Ω_dia_ and systolic geometry Ω_*sys*_ from image segmentation. (**B**) A nonlinear transform T from diastolic to systolic phase was obtained using non-rigid ICP registration^30^ and thin-plate spline (TPS) fitting^31^ algorithms, and therefore the distance between Ω_*sys*_ and the transformed geometry T(Ω_dia_) is minimized. (**C**) Diastolic and systolic quad meshes of the ATAA with mesh correspondence.

After image segmentation, each aorta geometry was represented by a triangular mesh. For each patient, since the diastolic aorta geometry Ω_*dia*_ and systolic aorta geometry Ω_*sys*_ have different numbers of nodes and elements, the displacement field from diastole to systole cannot be directly calculated. To obtain the displacement field, mesh correspondence between diastolic and systolic phases needs to be established. Herein, non-rigid ICP registration^30^ and TPS fitting^31^ algorithms were applied to find a nonlinear coordinate transform *T* from the template geometry Ω_*dia*_ to the target geometry Ω_*sys*_, such that the distance between Ω_*sys*_ and the transformed template geometry *T*(Ω_*dia*_) is minimized (Fig. 3(A,B)). Please refer to Amberg *et al*.^30^ for details of the registration method. The geometries of ATAA segment at diastolic phase were remeshed with quadrilateral elements (Fig. 1, yellow) using our previous remeshing algorithm^34^. Using the transform *T*, the quad ATAA meshes were transformed onto the surface of the ATAA segment at systolic phase (Fig. 3(C)). Thus, we obtained diastolic and systolic quad meshes of the ATAA with mesh correspondence.

### Constitutive model

Following our previous studies^25,26^, the Gasser-Ogden-Holzapfel (GOH) model^21^ was used to model the mechanical response of the aortic wall tissue. In this model, tissues are assumed to be composed of a matrix material with two families of embedded fibers, each of which has a preferred direction. The fiber directions can be mathematically described using two unit vectors. The strain energy function can be expressed by

1 $$\Psi ={C}_{10}({\bar{I}}_{1}-3)+\frac{{k}_{1}}{2{k}_{2}}\mathop{\sum }\limits_{i=1}^{2}[exp\{{k}_{2}{[\kappa {\bar{I}}_{1}+(1-3\kappa ){\bar{I}}_{4i}-1]}^{2}\}-1]+\frac{1}{D}[\frac{{J}^{2}-1}{2}-\,{\rm{l}}{\rm{n}}\,J]$$

where *C*_10_ is a material parameter to describe the matrix material. *k*_1_ is a positive material parameter that has the same dimension of stress, while *k*_2_ is a dimensionless parameter. The deviatoric strain invariant ${\bar{I}}_{1}$ is used to characterize the matrix material, and the deviatoric strain invariant ${\bar{I}}_{4i}$ is used to characterize the fiber families. ${\bar{I}}_{4i}$ is equal to squares of the stretches in the fiber directions.*κ* is used as a dispersion parameter describing the distribution of fiber orientation. When *κ* = 0, the fibers are perfectly aligned. When *κ* = 0.33, the fibers are randomly distributed, and the material becomes isotropic. *D* is a fixed constant enforcing the material incompressibility (*D* = 1 × 10^−5^). The mean fiber directions were assumed symmetric with respect to the circumferential axis of the local coordinate system. The parameter *θ* defines the angle between one of the mean local fiber direction and the circumferential axis of the local coordinate system. Thus, the five material parameters (*C*_10_, *k*_1_, *k*_2_, *κ*, *θ*) in this model need to be estimated.

### The inverse method for *in vivo* material parameter identification

**Figure 4:**
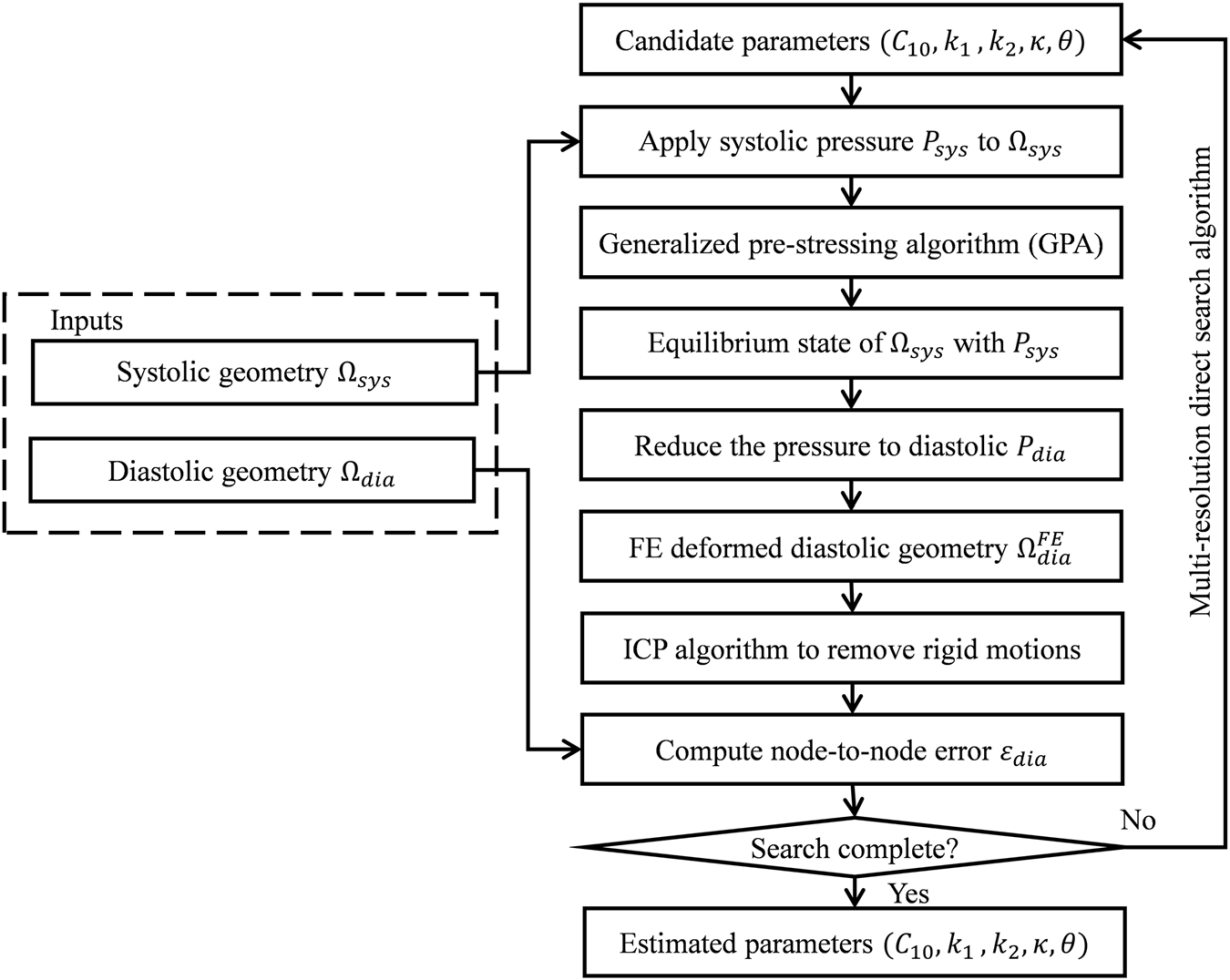
Workflow of the modified multi-resolution direct search (MRDS) approach.

We assume that the aortic wall is quasi-static at diastole and systole, respectively. The flowchart for constitutive parameter identification is demonstrated in Fig. 4. By using our modified multi-resolution direct search (MRDS) approach^25^ based on finite element (FE) updating, averaged material parameters throughout the ascending aorta were estimated from the *in vivo* systolic and diastolic ATAA geometries. Briefly, in the FE-updating scheme, with an initial guess of material parameters, (1) the pre-stresses associated with the systolic geometry are recovered by the generalized prestressing algorithm (GPA)^27^ implemented in FE simulation, (2) the geometry is depressurized to diastolic phase ${\Omega }_{dia}^{FE}$ in FE simulation, and (3) using the multi-resolution direct search (MRDS) strategy, the estimated material parameters are iteratively adjusted to minimize the average node-to-node error ε_*dia*_ between the FE-deformed diastolic geometry ${\Omega }_{dia}^{FE}$ and the *in vivo* CT-derived diastolic geometry Ω_*dia*_. This optimization process yields the optimal set of identified material parameters.

We define the objective function, which measures the average node-to-node error *ε*_*dia*_ between ICP registered ${\Omega }_{dia}^{FE}$ and Ω_*dia*_

2 $${\varepsilon }_{dia}({\Omega }_{dia}^{FE},{\Omega }_{dia})=\frac{1}{N}\mathop{\sum }\limits_{n=1}^{N}||{{\boldsymbol{X}}}_{dia,n}^{FE}-{{\boldsymbol{X}}}_{dia,n}||$$

where ${{\boldsymbol{X}}}_{dia,n}^{FE}$ is the coordinates of the *n*^th^ node on ${\Omega }_{dia}^{FE}$; *n* is the node index and *N* is the number of nodes; ||■|| denotes 3D Euclidean norm.

The FE simulations were performed in ABAQUS using C3D8H solid elements, and the mesh sensitivity analysis was performed in our previous work^35^. In the FE simulations, the boundary nodes were only allowed to move in the radial directions.

The node-to-node error ε_*dia*_ can be decomposed into two components: (1) rigid body motions (translation and rotation) and (2) strain. Due to the heart movements during the cardiac cycles (as can be observed in Fig. 3(C)), the boundaries of FE-deformed diastolic geometry ${\Omega }_{dia}^{FE}$ may not align with the boundaries of the *in vivo* CT-derived diastolic geometry Ω_*dia*_, and the rigid body motions between the two phases are not negligible. With the current boundary conditions, the rigid motions can result in large residuals in the objective function, and hence the error caused by strain could be overwhelmed. Thus, during each iteration of the material parameter identification, rigid ICP registration algorithm^28,29^ is applied to ${\Omega }_{dia}^{FE}$ to remove rigid motions with respect to Ω_*dia*_ before calculating the average node-to-node error ε_*dia*_.

In general, the GOH model^21^ has coupled material parameters, and the optimization problem in Eq. (2) is nonlinear, multivariate and non-convex, which causes the *in vivo* material parameter identification difficulty known as the local optima. Different combinations of *C*_10_, *k*_1_, *k*_2_, *κ* and *θ* in the GOH model may result in very similar mechanical responses. For the optimization methods that directly search the material parameter space, this coupling effect can produce numerous local optima. Gradient-based optimization approaches may not guarantee a global optimum. Mixed stochastic-deterministic approaches are typically used^23,24^ where material parameters are randomly initialized for the gradient-based optimization approaches. However, by directly searching the material parameter space, these approaches usually require a large number of iterations and may take weeks to complete^23,24^. In this study, we utilized our recently developed MRDS strategy^25^ to accelerate the optimization process.

In the MRDS approach^25^, a new search space, derived from the stress-stretch curves using principal component analysis (PCA), instead of the material parameter space, was used for the search of best material properties. The new PCA-based curve shape space is decomposed into multi-resolution representations, from coarse to fine. Instead of searching using the gradient of the objective function, the MRDS searches in the new PCA space at different resolutions and identifies the best curve shape match. Hence, to obtain the multiple resolution representations, the PCA space is represented by PCA parameter-candidates with multiple resolutions (note that PCA space parameters are different from the material parameters). In our previous study^25^, four resolutions were built: In the first, second, third and fourth resolutions, a total of 12, 117, 1197 and 10529 parameter-candidates were selected, respectively.

The parameter-candidates at one resolution are linked to the nearby parameter-candidates at adjacent resolutions according to the Euclidean distance. The MRDS strategy follows the links between multi-resolutions: a parameter-candidate with the lowest value of the objective function (Eq. (2)) is selected by searching the first resolution; following the links of the selected parameter-candidate, a new search begins at the next resolution. Consequently, the MRDS approach searches the discrete PCA space from the first (lowest) resolution to the last (highest) resolution, and the best parameter-candidate, which yields the lowest value of the objective function (Eq. (2)), can be eventually identified.

### Biaxial testing protocols

Stress-controlled biaxial tensile tests were performed on corresponding surgically-resected tissue samples of the two patients. Frozen tissue samples were submerged in a 37 °C water bath until totally defrosted, following the two-stage slow thawing method to remove the cryopreserving agent^36^. The samples were trimmed into 2~3 square-shaped specimens (2 specimens for Patient 1 and 3 specimens for Patient 2) with a side length of 20~25 mm. Each specimen was subjected to biaxial tension with the circumferential (θ) and longitudinal (*z*) directions aligned with the primary axes of the biaxial test fixture. A stress-controlled biaxial testing protocol was used^37,38^. *P* denotes the first Piola–Kirchhoff stress, and the ratio *P*_θ_:*P*_*z*_ was kept constant. Each tissue specimen was preconditioned for at least 40 continuous cycles with *P*_θ_:*P*_*z*_ = 1:1 to minimize tissue hysteresis. Seven successive protocols were performed using ratios ${P}_{{\rm{\theta }}}:{P}_{z}=0.75:1,0.5:1,\,0.3:1,\,1:1,\,1:0.75,\,1:0.5,\,1:0.3$. The GOH model parameters for each specimen were obtained by fitting the biaxial stretch-stress response in MATLAB. To obtain material parameters that approximately represent an average response, the stretch-stress data for all specimens from the same patient was fitted simultaneously.

### Ethical approval

The study was approved by the Georgia Tech and Emory University Institutional Review Boards.

## Results

### Wall thickness and blood pressures

The wall thickness values from *in vivo* and *ex vivo* measurements are shown in Table 1. Diastolic and systolic blood pressures for the two patients are reported in Table 2. The blood pressure levels were measured at the time of the patients’ visits for CT scans.

**Table 1:** Measured wall thickness (mean ± standard deviation) from CT scans and surgically-resected tissue.

Patient	Source	Wall thickness (mm)
1	*ex vivo*	2.29 ± 0.08
	*in vivo*	1.57 ± 0.60
2	*ex vivo*	1.95 ± 0.40
	*in vivo*	1.61 ± 0.37

**Table 2:** Diastolic and systolic blood pressure for the two patients.

Patient	Phase	Blood pressure (mmHg)
1	diastole	95
	systole	149
2	diastole	80
	systole	156

### *In vivo*-identified and *ex vivo*-fitted material properties

The improved MRDS approach can be completed in 1~2 hours with less than 100 FE iterations using a quad-core CPU with 32GB memory. The *in vivo*-identified and *ex vivo*-fitted material parameters are shown in Table 3, where the difference in material parameters can be clearly seen. The difference is anticipated mainly due to the highly coupled material parameters - leading to local optima in the optimization process (Section 2.3). The coefficient of determination (R^2^) values between the *ex vivo*-experimental and *ex vivo*-fitted stress/strain data are also reported in Table 3.

**Table 3:** *In vivo*-identified and *ex vivo*-fitted material parameters for the two patients.

Patient	Source	C_10_(*kPa*)	k_1_(*kPa*)	k_2_	κ	*θ*(°)	R^2^
1	*ex vivo* all	18.74	100.85	16.67	0.2800	0.00	0.95
	*ex vivo* 1	19.80	75.40	18.59	0.2809	0.00	0.95
	*ex vivo* 2	17.44	132.87	13.42	0.2812	0.00	0.96
	*in vivo*	45.00	50.00	50.00	0.2667	45.00	
2	*ex vivo* all	21.73	669.69	4.97	0.3200	34.28	0.58
	*ex vivo* 1	16.33	8.05	5.14	0.0000	0.00	0.93
	*ex vivo* 2	12.78	167.39	0.00	0.3035	90.00	0.96
	*ex vivo* 3	39.10	1157.64	0.00	0.3199	0.00	0.98
	*in vivo*	45.00	50.00	47.50	0.2667	30.00	

**Figure 5:**
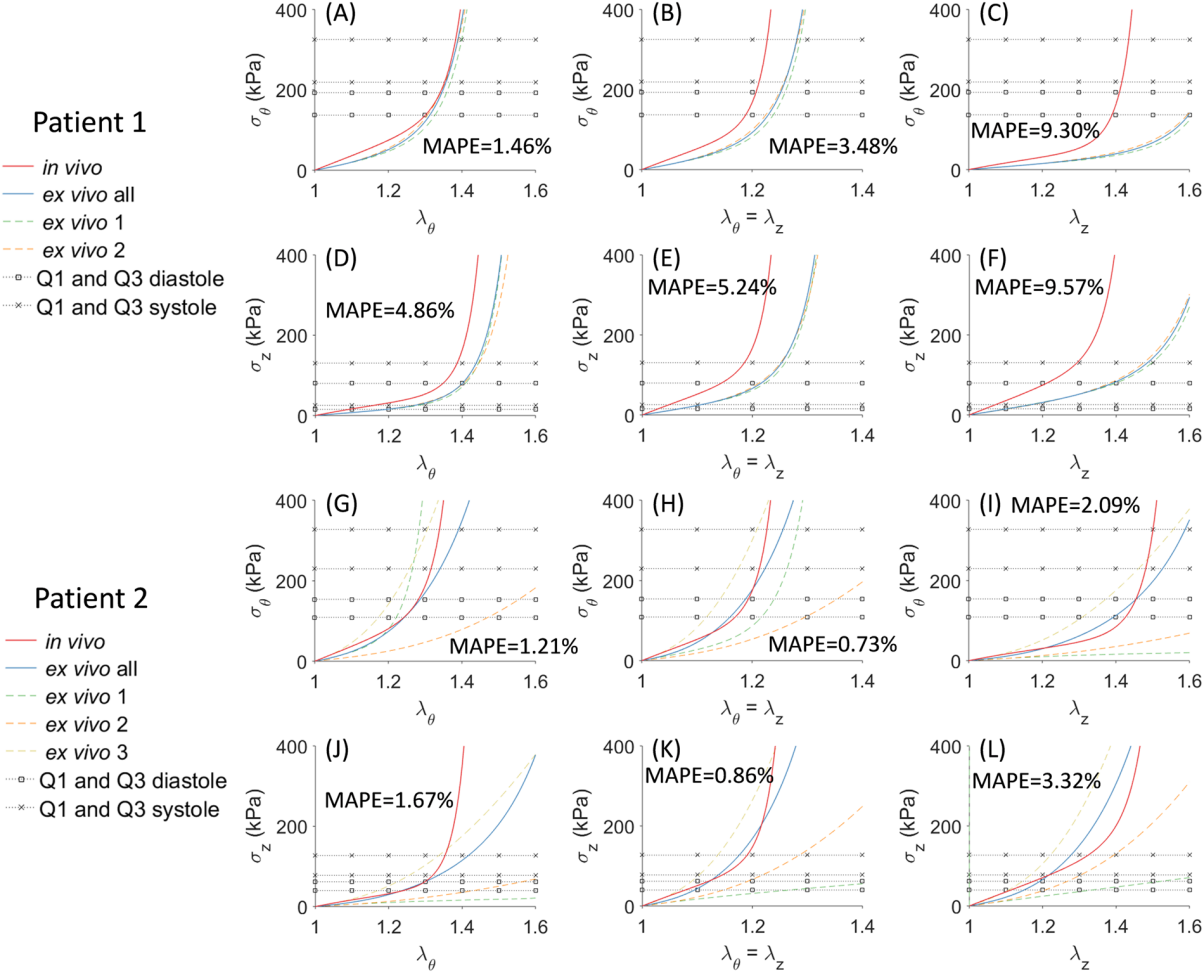
Stress-stretch curves determined from the *in vivo*-identified material parameters and *ex vivo*-fitted material parameters for Patient 1 ((**A**)~(**F**)) and Patient 2 ((**G**)~(**L**)). Left column: strip-biaxial protocol in the circumferential direction; middle column: equi-biaxial protocol; right column: strip-biaxial protocol in the longitudinal direction. First and third rows: circumferential stress, second and fourth rows: longitudinal stress. The average response is indicated by ‘*ex vivo* all’. Q1 and Q3 denote the 25% and 75% interquartile of *in vivo* stress range.

A more informative way for comparing material properties obtained from the inverse method and experiments, is to plot the stress-stretch curves computed from material parameters. We use *σ*_θ_ and *λ*_θ_ to denote the circumferential stress and stretch, *σ*_z_ and *λ*_z_ to denote the longitudinal stress and stretch. Thus, stress-stretch curves are plotted using the *in vivo-*identified *and ex vivo-*fitted material parameters with three *λ*_θ_:*λ*_z_ ratios, namely three protocols: (1) in the circumferential strip biaxial protocol, fixing *λ*_z_ = 1 while increasing *λ*_θ_; (2) in the equi-biaxial protocol, keeping the ratio *λ*_θ_:*λ*_z_ = 1:1; (3) in the longitudinal strip biaxial protocol, fixing *λ*_θ_ = 1 while increasing *λ*_z_. The stress-stretch curves determined by the estimated parameters were compared with the stress-stretch curves derived from biaxial data. As plotted in Fig. 5, the two specimens of Patient 1 demonstrate almost identical stretch-stress response, whereas the three tissue specimens of Patient 2 show different stress-stretch responses, which indicate that the material properties are heterogeneously distributed. Mean absolute percentage error (MAPE) is computed to measure the goodness-of-fit between *in vivo*-identified and *ex vivo-*fitted average curve (*ex vivo*-all). For both patients, the average response show relatively good agreements with the identified stretch-stress curves.

**Figure 6:**
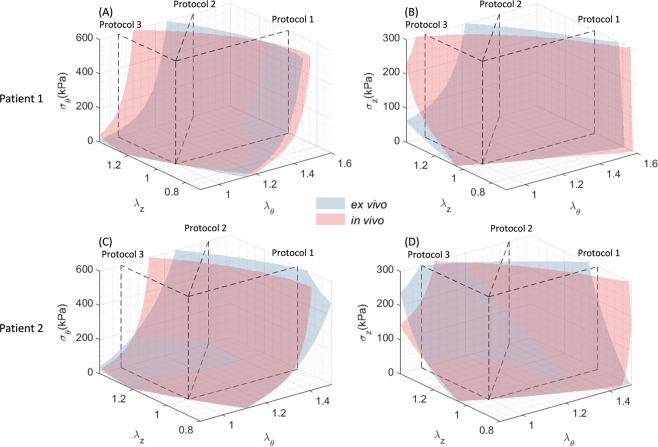
Stress-stretch response surfaces plotted using *in vivo*-identified and *ex vivo*-fitted material parameters. Left column: circumferential stress; right column: longitudinal stress. Dashed planes indicate the stretch-controlled biaxial protocols in Fig. 5: (1) strip-biaxial in the circumferential direction; (2) equi-biaxial; (3) strip-biaxial in the longitudinal direction.

To further compare the biaxial stretch-stress response, using the *in vivo*-identified material parameters, the stress *σ*_θ_ or *σ*_Z_ values can be obtained at various values of stretch *λ*_θ_ and *λ*_z_, which creates the response surfaces, by assuming a plane stress state and incompressible condition. Hence, the stretch-stress response surfaces for *in vivo*-identified and *ex vivo*-fitted average material response can be visualized in Fig. 6. Relatively close matches were achieved between the *in vivo*-identified and *ex vivo*-fitted response surfaces.

To compare linearized response in the circumferential direction within the physiological range, we computed pressure-strain modulus as defined in Martin *et al*.^5^ from image-derived geometries and FE-deformed geometries, respectively. *Ex vivo*-derived pressure-strain modulus can also be obtained using the same FE simulation setup (Section 2.3) with *ex vivo*-fitted average material parameters. The results are shown in Table 4, which show that close matches are achieved between the image-derived and FE-predicted the pressure-strain moduli. For Patient 2, the difference between FE-predicted and *ex vivo*-derived pressure-strain modulus may be explained by the deviation of curve slop in the physiological range (Fig. 5(G)).

**Table 4:** Image-derived, FE-predicted and *ex vivo*-derived pressure-strain moduli for the two patients.

Patient	Image-derived pressure-strain modulus (*kPa*)	FE-predicted pressure-strain modulus (*kPa*)	*Ex vivo*-derived pressure-strain modulus (*kPa*)
1	1433	1480	1582
2	1518	1529	789

### Comparison of the image-derived and FE-deformed geometries

**Figure 7:**
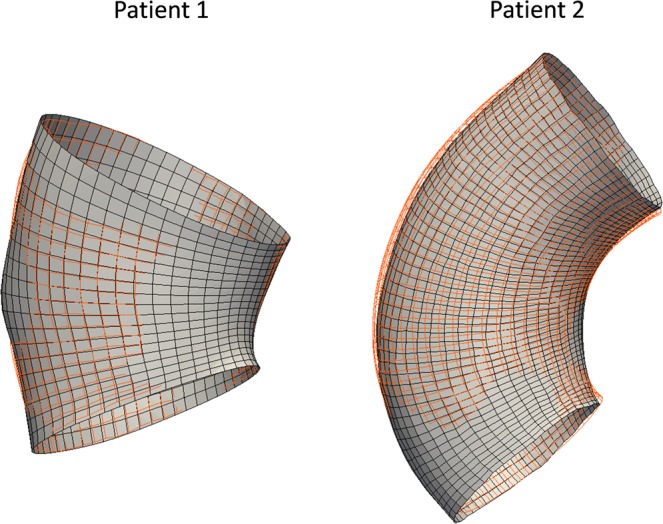
Image-derived and FE-deformed diastolic geometries (inner wall) for the two patients. Red wireframe: Image-derived; grey surface: FE-deformed.

The objective function (Eq. (2)) was minimized by the MRDS approach for the two patients, which measures the node-to-node error between the image-derived and the FE-deformed (using *in vivo*-identified material parameters) diastolic geometries. The image-derived and the FE-deformed diastolic geometries are plotted in Fig. 7. For comparison, we also compute the node-to-surface error defined by Eqs (2) and (3) of our previous work Liu *et al*.^25^. The errors are reported in Table 5. It is worth noting that, in general, the averaged node-to-node error is larger than the averaged node-to-surface error^25^.

**Table 5:** Node-to-node and node-to-surface errors of the image-derived and estimated diastolic geometries.

Patient	node-to-node error (Eq. (2)) (mm)	node-to-surface error^25^ (mm)
1	0.65	0.41
2	1.58	0.77

The relatively large value of node-to-node error for Patient 2 may be explained by heterogeneity of the material properties and wall thickness. In other words, deformations between the diastolic and systolic phases of Patient 2 cannot be fitted well using one set of material parameters. Hence, the diastolic-to-systolic displacement fields of image-derived and FE-deformed geometries are compared as follows.

**Figure 8:**
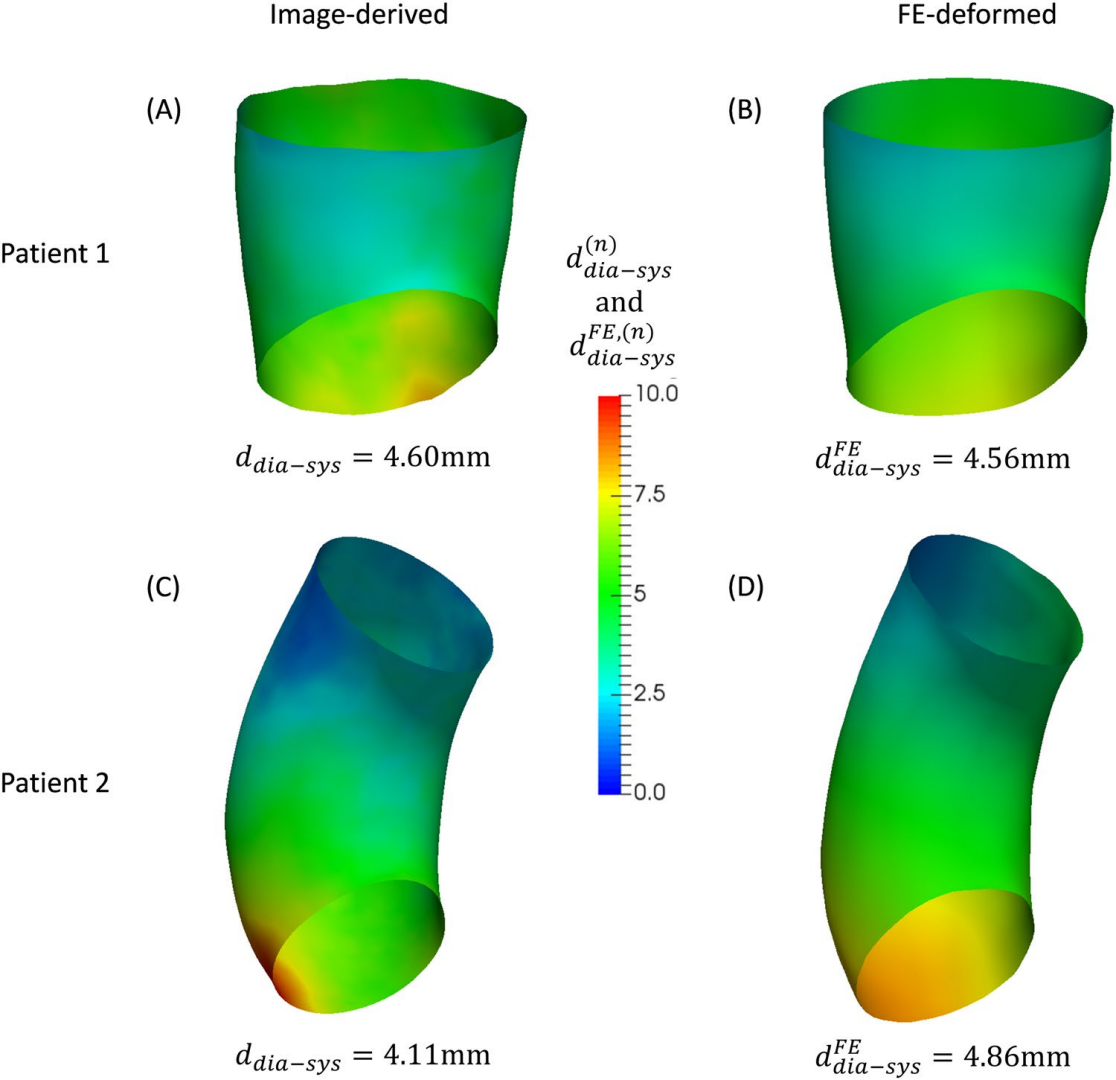
The displacement magnitude fields between diastolic geometries (image-derived: (**A**,**C**), FE-deformed: (**B**,**D**)) and the image-derived systolic geometries. The displacement magnitude fields are encoded as colors on the diastolic geometries.

For a node on the image-derived diastolic geometry Ω_*dia*_ and the corresponding node on the image-derived systolic geometry Ω_*sys*_, the displacement magnitude of a node is defined as

3 $${d}_{dia-sys}^{(n)}({{\boldsymbol{X}}}_{dia,n},{{\boldsymbol{X}}}_{sys,n})=||{{\boldsymbol{X}}}_{sys,n}-{{\boldsymbol{X}}}_{dia,n}||$$

where $\,{{\boldsymbol{X}}}_{sys,n}$ is the coordinates of the *n*^th^ node on Ω_*sys*_. The averaged displacement magnitude between the diastolic and systolic geometries, ${d}_{dia-sys}$ can also be obtained. Similarly, we can compute the FE-derived displacement magnitude ${d}_{dia-sys}^{FE,(n)}({{\boldsymbol{X}}}_{dia,n}^{FE},{{\boldsymbol{X}}}_{sys,n})$ between the FE-deformed diastolic geometry ${\Omega }_{dia}^{FE}$ and the image-derived systolic geometry Ω_*sys*_. Thus, the averaged displacement magnitude ${d}_{dia-sys}^{FE}$ can be calculated. The displacement fields for the two patients are plotted in Fig. 8. As expected, for Patient 2, the image-derived displacement fields are significantly heterogeneous comparing to the FE-derived displacement fields, while Patient 1 demonstrates only minor difference.

## Discussion

Despite the discrepancy in numerical values of the constitutive parameters, the inverse method and experiments achieved relatively good agreement in the biaxial stress-stretch curves and response surfaces. The averaged node-to-surface errors between the FE-deformed and the image-derived geometries were 0.41 mm and 0.77 mm for Patients 1 and 2, respectively, which are less than or equal to the size of a voxel (0.75 × 0.75 × 1 mm). This marks, to our knowledge, the first study that directly estimate *in vivo* nonlinear, anisotropic material properties of the ATAA from gated CT scans with comparisons to experimental data of planar biaxial tests. Trabelsi *et al*.^39^ utilized *in vitro* experimental bulge inflation test for inverse method validation. However, the study^39^ assumed isotropic constitutive behavior of ATAA and a linear relation between the aorta volume and the material parameters. In this study, the improved MRDS approach does not require such assumptions and can handle anisotropic tissue response.

The computational efficiency is further improved by the modified MRDS approach. This is mainly because that the computationally-expensive iterative method^34,40^ for recovering unloaded configuration is avoided by using the GPA method^27^. The improved MRDS approach can be completed in 1~2 hours with less than 100 FE iterations, using a quad-core CPU with 32GB memory, whereas the original MRDS method^25^ took 1~2 days using the same computation power.

The diastolic-to-systolic displacement field, which establishes mesh correspondence between diastolic and systolic phases, is often required by the material parameter identification schemes such as the virtual field method^41^ and the stress-matching^26,42^ or strain-matching method^23,24^. This requirement can be satisfied in *in vitro* experiments by tracking physical markers or tracking speckle patterns in ultrasound images^43^. CT is routinely used for imaging ascending aorta because of its large field of view^44^. However, CT images do not have distinct image texture patterns for tracking individual points on the aortic wall, and therefore the absence of diastolic-to-systolic displacement field poses a critical challenge for material parameter identification using CT data. In the original MRDS approach^25^, because the objective was based on geometry-matching, the use of node-to-surface objective function eliminates the need for mesh correspondence. In the improved version of MRDS, mesh correspondence was established using the non-rigid ICP registration^30^ and TPS fitting^31^ algorithms. Thus, the improved MRDS can support different identification schemes^24,26^ and material properties can be estimated from the gated CT data.

Because ECG-gated CT are not routinely performed for ATAA, we only acquired the multiphase CT data and tissue samples of two patients. In addition, only part of ATAA of Patient 1 was imaged from the multiphase CT data, so the identification are restricted on a small segment for Patient 1 (Fig. 1). Hence, the current inverse approach needs more validation cases before clinical application.

**Figure 9:**
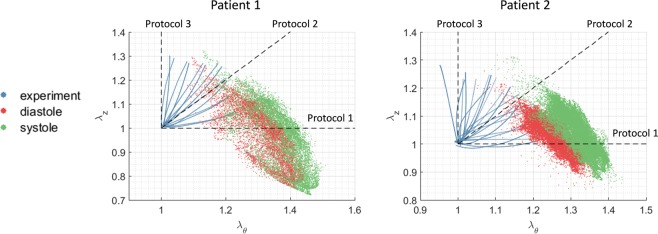
The experimental and physiological (diastolic and systolic) stretch values. Dashed line represent the stretch-controlled biaxial protocols in Fig. 5: (1) strip-biaxial in the circumferential direction; (2) equi-biaxial; (3) strip-biaxial in the longitudinal direction. The physiological stretch values were computed from the GPA method in Section 2.3. When the ascending aorta (“curved tube”) is pressurized, the inner curvature region expands radially towards the center of curvature, which can cause longitudinal shrinkage. Therefore, the axial deformation is in compressive mode at the inner curvature region.

The discrepancies between the *in vivo* identified and *ex vivo* fitted material parameter could be attributed to the following sources. (1) The experimental and physiological (diastolic and systolic) stretch values are shown in Fig. 9. The physiological stretch values (with pre-stretch/pre-stress considered) were computed from the GPA method in Section 2.3. It is worth noting that, the stretch ratios are different under experimental and physiological conditions, which indicates that the biaxial experimental data can only partially capture the physiological conditions. A constitutive model that can characterize the biaxial experimental data well may have to extrapolate its predication under some physiological conditions. (2) The aorta may undergoes rhythmic active contraction *in vivo* during the cardiac cycle^45^. However, active contractions generated by the smooth muscle cells are not considered by the constitutive model in this study. The surgically-resected tissues may only demonstrate passive *ex vivo* behavior. This could be a source of discrepancy between the *in vivo* and *ex vivo* properties. (3) The blood pressure levels were measured at the time of the patients’ visits for CT scans. Unfortunately, their blood pressures were not obtained simultaneously with the ECG-gated CT scans. (4) The external supports from pulmonary arteries and vena cava could alter the stress distribution within the aorta. Since the supporting forces are unknown, it could be a source of discrepancy between the *ex vivo* and *in vivo* properties. Note that there is no rigid (or high stiffness) structure (e.g. rib cage) that contacts the aorta, it is likely that the external supports from the pulmonary arteries/vena cava would not have significant impact on the loading bearing of the aorta. (5) In the FE simulations, the boundary nodes were only allowed to move in the radial directions. To validate this boundary condition, we plotted the FE-deformed (after ICP) versus image-derived diastolic geometries. From Fig. 7, close matches can be observed for the proximal and distal boundaries. (6) Heart motions could induce unknown axial forces/stresses in the aorta. However, using the current displacement boundary condition, the reaction forces at the proximal and distal ends required for the static equilibrium were calculated by the FEA. Because of different pressure loading conditions, the boundary forces are different for diastole and systole. Therefore, the axial stresses are also different at diastolic and systolic phases and are dependent on the patient-specific geometry and blood pressure levels.

The similarities of identified material parameters between the two patients could be explained by the similarities of the pressure-strain modulus (Table 4). It is unlikely that the similarities is due to constraint or initial values in the optimization. Unlike gradient-based optimization, in the MRDS, the parameter space is represented by a finite number of parameter-candidates prior to the optimization process. Therefore, the optimization always starts with the same parameter-candidates in the first level (12), and there is only a limited number of (10529) of possible solutions in the MRDS. The identified parameters were automatically determined by the MRDS algorithm. The upper and lower bounds used for sampling the parameter-candidates are shown in our previous study^25^, they are far from the identified parameters in this study. In addition, there is no bound or constraint during the MRDS optimization process.

In this study, the constitutive parameter identification was based on the following two main assumptions. (1) It is known that the aortic tissue properties are heterogeneously distributed^46,47^, material properties of the inner curvature region may be different from those of the outer curvature region. The wall thickness may also has spatial variation across the ATAA, heterogeneity of wall thickness and material heterogeneity could be correlated^48^. In the current inverse method, we only considered a simplified case, where the averaged *in vivo* wall thickness was used, and the averaged hyperleastic behavior of the aorta segment was identified. The stress-stretch data in this study (Fig. 5) also suggests material heterogeneity. The discrepancy in Figs 5 and 6 may be attributed to location-dependent material property distribution. As shown in Fig. 8, the displacement field explained by the FE simulation is smoother than the image-derived displacement field, which indicates an averaging effect when assuming homogenous properties. (2) We assume that residual stresses have minimal impact on the material parameter identification, since a study^49^ suggested that the inclusion of residual stress in the model has little effect on estimated material properties. Our recent work^50^ also demonstrated that the transmural mean stress is independent of the residual stress.

## Conclusion

To identify *in vivo* nonlinear anisotropic material properties of the ATAA, we have improved our original MRDS approach, thus computation time cost is further reduced and mesh correspondence can be established. The improved MRDS approach was applied to pre-operative gated CT scans of two ATAA patients. For comparison, surgically-excised tissue samples were obtained for experimental planar biaxial tests. Relatively close match was achieved in terms of the *in vivo*-identified and *ex vivo*-fitted stress-stretch response. Our results are preliminary, but encouraging. It is hoped that further development of this approach can enable an accurate identification of the *in vivo* material properties from gated CT data, which currently is a critical challenge in the field of cardiovascular biomechanics.

## Data Availability

All relevant data are within the paper.
